# Laser Capture Microdissection-Assisted Protein Biomarker Discovery from *Coccidioides*-Infected Lung Tissue

**DOI:** 10.3390/jof6040365

**Published:** 2020-12-14

**Authors:** Natalie M. Mitchell, Surendra Dasari, Thomas E. Grys, Douglas F. Lake

**Affiliations:** 1 School of Life Sciences, Mayo Clinic Collaborative Research Building, Arizona State University, Scottsdale, AZ 85259, USA; nmitche3@asu.edu; 2 Department of Health Sciences Research, Mayo Clinic College of Medicine, Rochester, MN 55905, USA; Dasari.Surendra@mayo.edu; 3 Department of Laboratory Medicine and Pathology, Mayo Clinic, Phoenix, AZ 85054, USA; grys.thomas@mayo.edu

**Keywords:** *Coccidioides*, Valley Fever, biomarker, mass spectrometry, laser capture microdissection

## Abstract

Laser capture microdissection (LCM) coupled to label-free quantitative mass spectrometry is a viable strategy to identify biomarkers from infected tissues. In this study, LCM was employed to take a “snapshot” of proteins produced in vivo during *Coccidiodies* spp. infection in human lungs. Proteomic analysis of LCM lung sections revealed hundreds of hosts and Coccidioidal proteins. Twenty-seven highly abundant *Coccidioides* spp. proteins were identified which do not share significant sequence orthology with human proteins. Three of the 27 Coccidioidal proteins are also potential *Coccidoides*-specific biomarkers, as they also do not share sequence homology to any other pathogenic fungus or microbe. Gene ontology analysis of the 27 biomarker candidate proteins revealed enriched hydrolase activity and increased purine and carbohydrate metabolism functions. Finally, we provide proteomic evidence that all 27 biomarker candidates are produced by the fungus when grown in vitro in a media- and growth-phase dependent manner.

## 1. Introduction

The fungal genus *Coccidioides* contains two species: *Coccidioides immitis* and *Coccidioides posadasii*, which are the etiological agents of coccidioidomycosis (Valley Fever; VF). *Coccidioides* spp. are dimorphic ascomycetes of the family Onygenaceae existing in a mycelial form in the soil (saprobic phase) and a yeast-like spherule form (parasitic phase) in infected mammalian hosts [1]. There is no single serological assay for VF that serves as an ideal diagnostic assay, and all available methods have moderate sensitivity for detection of antibodies in the first few weeks. Sensitive detection of specific *Coccidioides* spp. antigens released from spherules in the acute phase of Valley Fever infection (VF) could be the ideal approach for diagnosis of VF. Detection of antigen in a body fluid would allow for early and definitive laboratory diagnosis of VF. However, the only antigen assay commercially available has moderate sensitivity, cross-reacts with other fungi, and must be sent to a reference laboratory [2]. Thus, there is much work to be done to truly advance the goal of early and accurate diagnosis of coccidioidomycosis.

With over 3500 plasma proteins in the Human Proteome Organization (HUPO) human plasma proteome database [3,4] and an estimated >500 proteins circulating at any one time in human plasma [5], identification of low abundance biomarker proteins among high abundance plasma proteins like albumin and immunoglobulins is a difficult challenge in mass spectrometry-based biomarker discovery [5,6]. To be able to identify low abundance proteins, enrichment using selective antibodies or deletion of highly abundant proteins from serum using other affinity reagents are frequently employed options. However, in order to identify the correct affinity enrichment reagent or to produce a selective antibody, one must identify and produce target biomarkers.

Identification of proteins from pure cultures of an infectious microorganism growing in vitro is not difficult. However, the most abundant proteins produced during in vitro culture may not be fully representative of the in vivo proteome of organisms, particularly in the case of a dimorphic fungus such as *Coccidioides*. Protein production is known to be influenced by a number of host pressures not available in vitro (immune cells, adhesion to host tissues, hypoxia, nutrient gradients/deprivation, osmotic stress, etc.) These factors likely explain the significant differences in phenotype, virulence, and survival that have been noted between fungi grown in vivo versus in vitro [7,8]. Additionally, even different in vitro culture media formulations can cause significant phenotypic changes in *Coccidioides* spp. [9].

One biomarker discovery platform which is able to evaluate protein abundance in vivo is laser capture microdissection (LCM) followed by mass spectrometry. LCM facilitates the sampling of selected cellular structures and regions from ex vivo tissues. Here we describe and reveal results of an LCM-assisted label-free quantitative proteomic technique for the identification and relative quantification of VF protein biomarkers from extracted *Coccidioides* spherules from lung biopsies. In an effort to identify in vitro growth conditions that recapitulate growth conditions in human lungs, protein identifications and abundances from in vivo spherules were compared to the protein abundances produced from in vitro-grown spherules and mycelia in different media and conditions.

## 2. Materials and Methods

### 2.1. *Coccidoides* spp.-Infected Tissue Samples

Triplicate technical replicates of archived formalin-fixed paraffin embedded (FFPE) lung tissue blocks from each of 3 naturally infected human clinical cases were used in this study. Infections had been culture-confirmed cases of *Coccidioides* spp., and all three patients were immunosuppressed (2 HIV positive individuals and 1 treated with adalimumab). Tissue blocks were acquired from Mayo Clinic Arizona histology biobank in accordance with IRB# 12-000965 (human tissue). Ten micrometer thick sections were cut using a microtome and transferred to 1 mm polyethylene naphthalate (PEN) membrane slides (Carl Zeiss Microscopy; Jena, Germany), deparaffinized and stained with hematoxylin and eosin stain (H&E) using standard procedures.

### 2.2. LCM of FFPE Tissues

**Figure 1:**
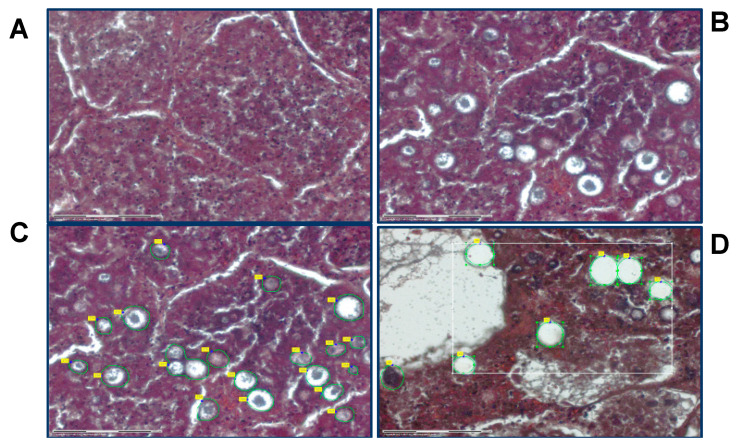
Laser capture microdissection (LCM) of *Coccidioides* spp.-infected human lung tissue is able to precisely extract spherules. Clear (white) circles rimmed with green and with yellow boxes indicate areas where spherules were laser-captured. Tissue sections without fungal spherules (**A**) can be ignored, whereas sections with spherule elements (**B**) can be selected (**C**) and exclusively removed (**D**).

Laser capture microdissection (LCM) was performed using a Zeiss PALM MicroBeam scope with RoboPalm software (Carl Zeiss Microscopy). Approximately 500,000 μm^2^ area of spherules in lung tissue was collected for each sample by laser capture. Tissue features (spherules) were selected, collected and catapulted into the cap of 0.5 mL Eppendorf tubes containing 35 µL of 0.1 M Tris-HCl (pH = 8.0), 0.002% Zwittergent Z3-16 (MilliporeSigma, Burlington, MA, USA) via laser pressure catapulting (Figure 1). Immediately after capture, the tube was centrifuged at 14,000× *g* for 2 min to collect the lysis solution and tissue into the bottom of the tube and was frozen at −80 °C until processing.

### 2.3. In-Solution Protein Digestion

Formalin–protein crosslinks were broken from the tissue fragments by heating the sample at 99 °C for 1 h. Proteins were reduced by the addition of 1.8 µL of 200 mM tris(2-carboxyethyl)phosphine (TCEP, MilliporeSigma) in 0.1 mM Tris, pH 8.2, (final conc. TCEP = 10 mM) and incubated at 56 °C for 30 min. Afterwards, proteins were alkylated by the addition of 1.9 µL of 200 mM iodoacetamide (IAA, MilliporeSigma) in 0.1 M Tris-HCl (final conc. = 10 mM) and incubated in the dark at RT for 30 min. Finally, proteins were digested by adding 2 μL of 0.01 µg/µL trypsin (Thermo, Waltham, MA, USA) and incubated at 37 °C for 16 h. The digestion was terminated by adding 4 μL of 2% formic acid (FA) prior to loading on the mass spectrometer. Loading was normalized to the area of tissue, optimally 500,000 square microns per sample provides ~500 ng protein.

### 2.4. Fungal Culture Preparation and Protein Lysis

The mycelial form of *Coccidioides posadasii* strain Silveira (strain kindly provided by Dr. Bridget Barker of the University of Arizona) was grown in a shake flask rotating at 180 RPM in 30 °C for 7 days in one of the following six liquid medias: (1) Levine modification of Converse media [10] with 0.5% N-Tamol (Dow Chemical Company, Midland MI), (2) RPMI-1640 medium (Corning) with 10% fetal bovine serum (FBS) or (3) RPMI-1640 medium with 0.1% Survanta (Beractant bovine lung surfactants; Abbot Laboratories, Chicago, IL, USA).

Spherule form of *C. posadasii* strain Silveira cultures were grown in vented shake flasks rotating at 180 RPM in 20% CO_2_ at 40 °C for 7 days in the same three media as above. Cultures were centrifuged at 4500 RPM for 30 min. in a Thermo Forma Multi RF tabletop centrifuge (Thermo). Supernatants were then sterilized by filtration through 0.45µm vacuum filtration units (MilliporeSigma). Culture pellets were resuspended 5:1 in fungal lysis buffer (50 mM Tris, pH 7.6, 100 mM NaCl, 50 mM EDTA, 5% SDS) prior to transfer into 1.5 mL silicone washer-sealed internally threaded cryovials containing ~350 µL of 0.5 mm acid-washed sterile glass beads. Spherule culture pellets in cryovials were subjected to two rounds of bead beating at maximum speed for 15 min followed by 3 cycles of flash freezing on dry ice. Sterility/viability was checked by plating 10% of the total volumes onto 2× glucose yeast extract agar (2 × GYE) for 3 weeks at RT.

### 2.5. Fungal Culture Protein Extraction and In-Gel Protein Digestion

Fungal culture proteins from filtered supernatants and pellet lysates were extracted using a modification of a previously published protocol [11]. In brief, supernatants and pellets were centrifuged for 30 min. at 8000 RPM at 4 °C. The supernatants were put into fresh tubes and proteins were precipitated by the addition of 4 volumes of ice cold 10% *w/v* trichloroacetic acid (TCA, MilliporeSigma) in acetone with 0.007% *w/v* dithiothreitol (DTT, G Biosciences, St. Louis, MO, USA). Samples were centrifuged at 3000× *g* for 10 min., and the resultant protein pellets were washed three times in ice cold acetone with 0.007% DTT. The final pellet was then resuspended in rehydration buffer [7 M urea, 2 M Thiourea, 4% *w/v* 3-[(3-Cholamidopropyl)dimethylammonio]-1-propanesulfonate hydrate (CHAPS, all MilliporeSigma) and 20 mM DTT].

Samples in rehydration buffer were then filter exchanged with phosphate buffered saline (PBS), using Amicon^®^ Ultra 0.5 mL 3 kDa filtration units (MilliporeSigma) prior to protein estimation using a micro bicinchoninic acid (BCA) protein assay kit (Thermo), and ~10 µg of protein from the supernatant samples and ~30 µg of corresponding pellet proteins were mixed with reducing Laemmli sample buffer (BioRad, Hercules, CA, USA), heated at 95 °C for 5 min. prior to loading on mini-Protean™ TGX precast gels (BioRad). Therefore, a total of 40 µg of combined supernatant and pellet proteins per sample were run in each sample lane. Following electrophoresis, gels were stained with Bio-Safe Coomassie G-250 Stain (BioRad) as per manufacturer’s instructions. Each sample lane of the SDS-PAGE gel was cut into six equal size slices. Band slices were then further reduced into cubes of 1–2 mm^3^ and put into low protein binding tubes (Eppendorf, Hamburg, Germany) prior to destaining. Proteins were reduced in 10 mM DTT for 30 min. at 60 °C, and alkylated with 55 mM ioadacetamide (IAA, MilliporeSigma) for 30 min. at room temperature in the dark, prior to 37 °C overnight digestion with Pierce™ MS Grade trypsin protease (Thermo) diluted to 20 ng/mL in 100 mM ammonium bicarbonate (MilliporeSigma). The peptides were then extracted from the gel pieces using 5% FA, ammonium bicarbonate and acetonitrile washes prior to being dried in a speed vacuum and stored at −80 °C until LC-MS analysis.

### 2.6. Proteomic Analysis of Fungal Pellets and Supernatants

Protein digests were reconstituted in 0.1% FA and analyzed using LC-MS/MS by loading onto a Dionex UltiMate^®^ 3000 RSLC liquid chromatography (LC) system (Thermo, San Jose, CA, USA) using a PepMap RSLC C18 2 um, 75 um × 50 cm EASY-Spray™ column (Thermo). Peptides were separated using a 0.3 uL/min LC gradient comprised of 2–90% mobile phase B in 0–120 min. Mobile phase A and B were 0.1% FA in water and acetonitrile, respectively. Eluting peptides were directly injected into an Orbitrap Elite Velos mass spectrometer (Thermo) and ionized using collision-induced dissociation (CID) in positive ion mode. A “top 15″ data-dependent MS/MS analysis was performed (acquisition of a full scan spectrum followed by collision-induced dissociation mass spectra of the 15 most abundant ions in the survey scan).

### 2.7. Protein Identification and Label-Free Protein Quantification

Database searching was performed using Sequest (Thermo) in Proteome Discoverer v1.4.1.14 (Thermo) against a combined FASTA database of all the most recent Uniprot *Coccidioides* spp. proteomes (*Coccidioides immitis* RS, proteome ID: UP000001261, 12 June 2018 release date; *Coccidioides immitis* RMSCC 3703, proteome ID:UP000054559, 26 February 2018 release date; *Coccidioides immitis* H538.4, proteome ID: UP000054563, 26 February 2018 release date; *Coccidioides immitis* RMSCC 2394, UP000054565, 26 February 2018 release date; *Coccidioides posadasii* strain RMSCC 757/Silveira, proteome ID: UP000002497, 26 February 2018 release date, *Coccidioides posadasii* C735, proteome ID: UP000009084, 9 November 2018 release date; and *Coccidioides posadasii* RMSCC 3488, UP000054567, 26 October 2018 release date). Searches were performed using a fragment tolerance of 0.60 Da (Monoisotopic), parent tolerance of 10 ppm (Monoisotopic), with carbamidomethyl of cysteine as fixed and oxidation of methionine as variable modifications with maximum missed cleavages allowed of 2. Protein identifications were accepted if they achieved a minimum of 2 peptides per protein and a false discovery rate (FDR) of <1%. Label-free protein quantification was performed using normalization of spectral abundance factors (NSAF) in Scaffold (v4.8.7, Proteome Software Inc., Portland, OR, USA).

### 2.8. Biomarker Identification and Bioinformatics

The top 100 most abundant *Coccidioides* spp. proteins found in LCM lung tissues from the mean of triplicate technical replicates were identified. These “top 100” most abundant Coccidioidal proteins were subjected to a pBLAST search (www.blast.ncbi.nlm.nih.gov) against the *Homo sapiens* proteome (taxid:9606), to exclude human orthologues. An independent analysis was performed to detect *Coccidioides* spp. peptides found in the LCM lung tissues and assess their homology against any peptides found in human and non- *Coccidioides* spp. fungi proteins present in NCBI non-redundant (NR) database. Detected peptides were filtered to retain *Coccidioides* spp. peptides that do not share 100% homology with either human or non- *Coccidioides* spp. fungi proteins (Supplementary Table S2). These peptides could be utilized to develop targeted mass spectrometry-based assays for detecting the presence of *Coccidioides* spp. in human biological samples.

Gene ontology identifiers of each remaining protein were pulled from Uniprot (www.uniprot.org). A gene ontology enrichment scatterplot was produced using the Revigo [12] plugin in FungiDB (www.fungidb.org) [13]. The wordcloud of enriched KEGG metabololic pathways was produced using the GOSummaries [14] plugin, also from FungiDB. Protein O-glycosylation predictions were made using NetOGlyc server 4.0 [15]. N-glycosylation and signal peptide predictions were determined using NetNGlyc 1.0 server [16].

### 2.9. Statistical Analyses

GraphPad Prism v.8.0 was used for production and statistical analyses, including those used in creating volcano plots and box and whisker plots. Volcano plots were created using multiple t tests of Normalized Spectral Abundance Factor (NSAF), corrected for multiple comparisons using Holm–Sidak method. NSAF is a unitless, arbitrary value used to rank abundance of proteins across samples and experiments [17]. Analysis of in vivo versus in vitro grown spherule abundances was calculated using a one-way ANOVA followed by Tukey test and adjusted for multiple comparisons using Sidak’s method.

## 3. Results

### 3.1. Proteomic Analysis and Biomarker Selection

**Figure 2:**
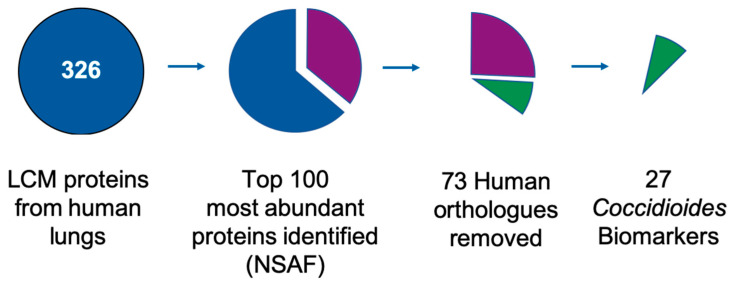
Laser capture microdissection (LCM) assisted biomarker discovery flowchart. There were a total of 326 *Coccidioides* spp. proteins identified in infected human lung tissues by mass spectrometry. Proteins with the 100 highest normalized spectral abundance factors (NSAFs) were further evaluated for orthology to human proteins (pBLAST > 40% identity). Seventy-two human orthologues were removed, leaving 27 *Coccidioides* spp. biomarker candidates.

Using a 1% FDR cut-off there were a total of 326 *Coccidioides* spp. proteins identified by mass spectrometry from the lung tissue samples used in this study (Figure 2). After *Coccidioides* spp. protein identities were established, they were then analyzed for possible biomarkers. The mean normalized spectral abundance factors (NSAF) of triplicate technical replicates were calculated and the top 100 most abundant proteins were identified.

In order to identify the best proteins for use in an antigen-based detection method, seventy-three *Coccidioides* spp. proteins with human orthologues (pBLAST ≥ 40% identity) were removed from the dataset, leaving 27 possible *Coccidioides* spp. biomarkers. A pBLAST search of these 27 possible biomarker proteins against all fungi (taxid: 4751) revealed between 53% and 93% shared identity with other pneumonia-causing fungal species for 24/27 of the biomarker candidates. Only 3 proteins did not share significant identity with other pneumonia-causing fungal species; of which all 3 of these proteins were uncharacterized proteins (CIMG_00509, CIMG_05576, CIMG_09001), and shared sequence identity in *C. immitis* and *C. posadasii* strains.

**Figure 3:**
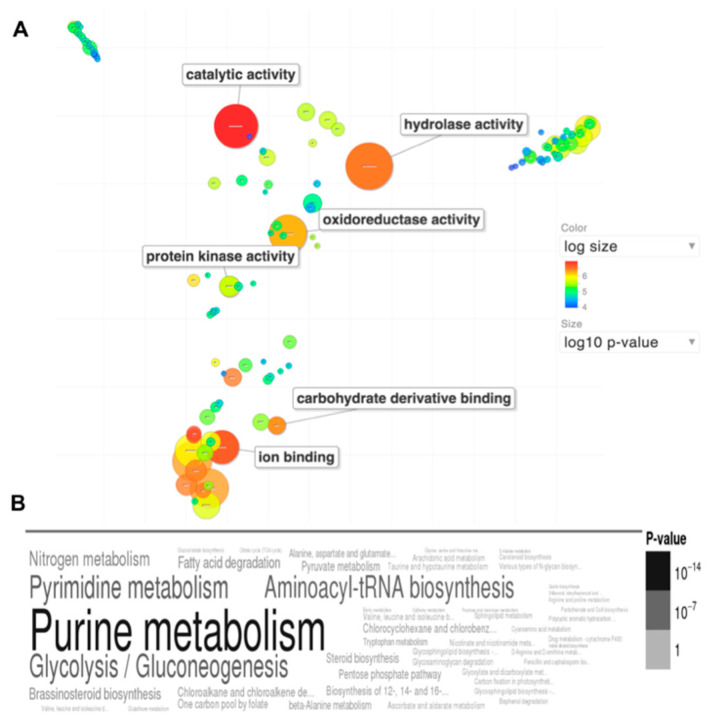
Gene ontology enrichment analysis of the 27 biomarker candidates indicates enriched hydrolase activity and increased purine and carbohydrate metabolism functions. (**A**) Scatterplot of gene ontology enrichment of molecular functions, with size of the circles is proportional to the significance of enrichment. (**B**) Word cloud of enriched KEGG metabolic pathways. Size of the words indicates significance of enrichment. Figures were produced using the Revigo (Supek and others 2011) and GOSummaries (Kolde and Vilo 2015) wordcloud plugin from FungiDB (www.fungidb.org) (Basenko and others 2018).

Gene ontology enrichment analysis of the 27 biomarker candidates indicates significant increases in certain molecular functions and metabolic pathways. As seen in Figure 3A, catalytic, hydrolase and oxidoreductase activities are most increased, with moderate increase in carbohydrate derivative binding. Enrichment of KEGG metabolic pathways in Figure 3B indicate increased purine/pyrimidine metabolism, as well as glycolysis/gluconeogenesis.

The 27 biomarker candidate proteins were then identified from in vitro mycelial and spherule forms of fungal cultures, each in three different media preparations (Table 1). Moreover, included in this table is information regarding protein molecular weight, protein glycosylation, and secretion signal predictions. The mean NSAF of triplicate technical replicates for all biological replicates (n) are provided in Supplementary Table S1. Of the 27 biomarker candidates, 13 (48.1%) were predicted to contain N-glycosylation sites, 18 (66.7%) were predicted to contain O-glycosylation sites, and 7 (25.9%) were predicted to contain signal peptides. All 27 biomarker candidates are less than 65 kDa in size, with 23 (85.2%) at being <45 kDa in size, of which 10 (37.0%) are ≤20 kDa in size.

### 3.2. Protein Abundances In Vitro and In Vivo

At some abundance level, all 27 (100%) biomarker candidates were expressed in vitro in at least one culture medium and growth phase combination (Supplementary Table S1). However, 1 of the biomarker candidates (Uncharacterized protein CIMG_09001) was not present in in vitro grown spherules in any of the 3 media used, and only in two of the mycelial culture media. Likewise, 2 of the biomarker candidates (Cytochrome c oxidase polypeptide VI and Uncharacterized protein CIMG_05576) were not present in any of the in vitro grown mycelial cultures but were present in in vitro grown spherules. With regards to the in vitro growth of spherules, the media that produced the most biomarker candidate proteins 26/27 (96.3%) was RPMI + Survanta, with Converse + Tamol producing 23/27 (85.2%) and RPMI + FBS produced 21/27 (77.8%) of the biomarker candidates. For the mycelial form, the media that produced the most biomarker candidate proteins was RPMI + FBS, which produced 24/27 (88.9%) of the proteins, whereas Converse + Tamol and RPMI + Survanta only produced 18/27 (66.7%) of the proteins in the mycelial form.

**Figure 4:**
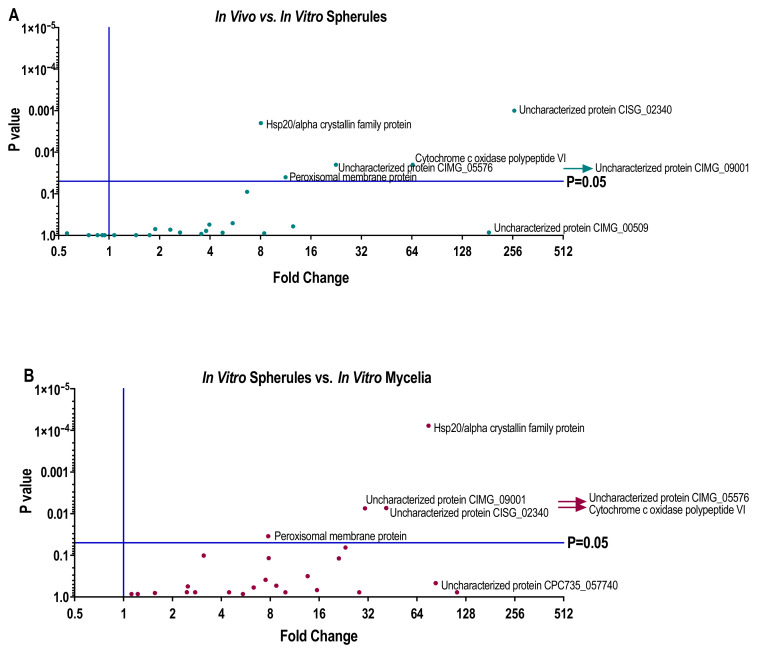
Volcano plots of the 27 biomarker candidate proteins indicate large fold change of protein abundances (NSAF) between in vivo and in vitro grown proteins. (**A**) Fold change of relative protein abundances of LCM lung tissues in relation to the spherule form. (**B**) Fold change of relative protein abundances in the spherule form in relation to the mycelial form. Proteins next to arrows had a significant *p* value, but as they did not grow in vitro (had NSAF value of 0), could not calculate a fold change. Volcano plots produced in GraphPad Prism v.8, using multiple t tests of NSAF, corrected for multiple comparisons using Holm–Sidak method.

The mean NSAF abundances in Supplemental Table S1 were used to calculate protein abundance fold changes between in vivo spherules and in vitro grown spherules and mycelia. In Figure 4A, a volcano plot shows fold change of protein abundance (NSAF) of the 27 proteins from in vivo spherules relative to the protein abundance from in vitro spherules. Six of the 27 biomarker candidate proteins were significantly more abundant in the in vivo spherules than in vitro grown spherules: Hsp20/alpha crystallin family protein, Peroxisomal matrix protein, Cytochrome c oxidase polypeptide VI, Uncharacterized protein CISG_02340, Uncharacterized protein CIMG_09001, and Uncharacterized protein CIMG_05576. The fold change for Uncharacterized protein CIMG_09001 could not be calculated as this protein was not present at all in in vitro spherule cultures; however, the highest calculable fold change was Uncharacterized protein CISG_02340 (~261 fold greater in vivo than in in vitro spherules).

Fold change of relative protein abundances from in vitro grown spherules in relation to in vitro grown mycelia is shown in Figure 4B. Six proteins were significantly more abundant in the in vitro grown spherules than in vitro grown mycelia: Hsp20/alpha crystallin family protein, Peroxisomal matrix protein, Cytochrome c oxidase polypeptide VI, Uncharacterized protein CISG_02340, Uncharacterized protein CIMG_09001 and Uncharacterized protein CIMG_05576. The protein with the greatest calculable fold change was mitochondrial ATP synthase delta chain (~113 fold greater in spherules), although this difference was not statistically significant.

**Figure 5:**
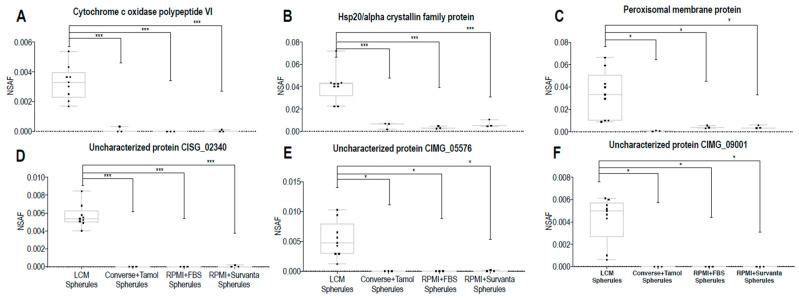
Box and whisker plots of differential protein abundances indicate that all biomarker candidate proteins can be produced in vitro, but abundance is dependent on culture media used. In vivo versus in vitro grown spherule abundances are shown for 6 proteins with the highest fold changes (**A**–**F**). Significance was calculated using one-way ANOVA adjusted for multiple comparisons using Holm–Sidak method (* *p* < 0.05, *** *p* < 0.001).

The most significantly abundant proteins from Figure 4A,B were then evaluated for their ability to be produced in vitro. Figure 5A–F shows the protein abundances of each in vitro media used, as compared to the abundance of the same protein in vivo. All of the 6 most significantly abundant in vivo spherule proteins were able to be produced in spherules in vitro, however abundances were media dependent. For example, CIMG_05576 was more abundant in spherules grown in RPMI + Survanta than it was in other growth conditions, including mycelial cultures, but was still 147-fold lower abundance than it is when spherules grow in human lungs.

## 4. Discussion

In this study, biomarker candidate proteins were identified from *Coccidioides* spp.-infected lung tissue using LCM coupled with mass spectrometry. The 100 most abundant proteins in LCM-captured spherules were identified, and their expression in vitro was measured in three different culture media conditions. Of the one-hundred, 73 human orthologues sharing ≥40% identity to human proteins were removed, leaving 27 possible *Coccidioides* spp. biomarker proteins. All 27 (100%) of the biomarker candidate proteins were expressed in vitro in at least one growth phase-medium combination; however, protein abundance depended on both growth phase and medium type. Additionally, three of the 27 biomarker candidates were found to be *Coccidioides*-specific biomarkers (Uncharacterized proteins CIMG_00509, CIMG_05576, CIMG_09001), as they do not share significant sequence identity to other fungal lung pathogens. Subsequent studies may help characterize the functions of these proteins and further determine if they are unique among all fungal pathogens.

Limitations of this study include the low number (n = 3) different human lungs tissue from which we performed LCM-mass spectrometry. Although this number is low, it likely represents more host-pathogen interaction diversity than examining infected lungs from 12 inbred mice. To date, there has only been one other proteomic evaluation of *Coccidioides* spp. proteins from in vivo produced samples. In this study by Lewis et al. (2015), who evaluated *Coccidoides* spp. proteins collected from bronchiolar lavage fluid (BALF) of laboratory-infected mice [18], only 8 *Coccidioides* spp. proteins were identified. In fact, all 8 of the *Coccidioides* spp. proteins identified in BALF were also identified in our LCM lung tissue dataset. Remarkably, 2 of the 8 (25%) BALF proteins they identified (Peroxisomal membrane protein and Uncharacterized protein CIMG_09001) were in our 27 candidate biomarkers dataset. However, the other 6 proteins they identified in BALF either shared too much identity with human proteins or were not in the top 100 most abundant proteins from LCM lung tissues. It is interesting that Uncharacterized protein CIMG_09001, which we identified as a *Coccidioides*-specific biomarker candidate, was produced in BALF. This data gives credence to the LCM-assisted biomarker discovery methodology used in this study.

Barring the Lewis et al. study, all other published proteomic studies of *Coccidioides* spp. have evaluated proteins produced from in vitro cultures, which are not truly representative of in vivo expression [4,19,20,21]. Additionally, all previous studies used only one culture medium: Converse media supplemented with Tamol. Interestingly, of the 27 biomarker candidates identified in this study as abundantly produced in vivo, 4 (14.9%) were not produced in the spherule form, and 9 (33.3%) were not produced in the mycelial form, when grown in Converse with Tamol in vitro. Previous studies which only used in vitro growth in Converse are likely to have missed these proteins. Other factors which may result in different findings include species and provenance of the *Coccidioides* isolate, as well as protein extraction methods employed.

One of the aims of this study was to find a single growth medium which causes spherule protein expression to most closely resemble in vivo growth. However, none of the media tested closely mimics in vivo growth as spherules. Different in vitro culture media and conditions allowed for different subsets of *Coccidioides* proteins to be produced. Regardless, of the 3 media evaluated in this study, the maximum number of the 27 biomarker candidates was produced using RPMI+ Survanta (26/27; 96.3%) in spherule cultures. RPMI media supplemented with Survanta was recently published by our group for in vitro culture of *Coccidioides* spp. [22]. We chose Survanta as an alternative surfactant to Tamol, which is a synthetic anionic surfactant made from sulfonic acid salts, more commonly used as a paint dispersant. Tamol has been shown to induce faster spherulation in in vitro culture and cause reversion of mycelia into spherules [23,24]. Although the exact mechanism of Tamol-induced spherulation is not entirely known, it is possibly due to its ability to emulsify media components and reduce surface tension around the spherule cell walls. Survanta is a natural bovine lung extract used in the prevention and treatment of respiratory distress syndrome in premature infants. In addition to containing surfactant proteins B and C (SP-B and SP-C), it contains phospholipids, neutral lipids, fatty acids, and surfactant-associated proteins which mimic the surface-tension lowering properties of natural lung surfactant. Additionally, as a working solution of 0.1% Survanta in RPMI, it contains only 0.1 mg/mL protein, and is much more compatible with mass spectrometry than RPMI supplemented with 10% FBS. While RPMI with Survanta is not the ideal in vitro proxy to represent spherule growth in the lung, our results in protein expression and glycan expression [22] suggest that further investigation of media types could yield further insights into the biology of *Coccidioides* spp.

In vivo grown spherules also produced protein abundances that differed from in vitro grown spherules. Peroxisomal matrix protein, Hsp20/alpha crystallin family protein, Cytochrome c oxidase polypeptide VI, Uncharacterized protein CISG_02340, Uncharacterized protein CIMG_09001, and Uncharacterized protein CIMG_05576 were all significantly more abundant in vivo. While the function of the Uncharacterized proteins is unknown, small heat shock proteins like the Hsp20/alpha crystallin family protein have been implicated in the pathogenesis of the plant fungal pathogen *Ustilago maydis* [25], as well as the pathogenicity and persistence of the bacterial lung pathogen *Mycobacterium tuberculosis* [26]. Peroxisomal matrix protein, in addition to being found in mouse BALF, has also been investigated as a recombinant vaccine candidate for VF [4,27]. Although it showed protection against intraperitoneal challenge, only modest protection was afforded following high dose intranasal challenge. In addition to being biomarker candidates, any of the other 27 proteins have the potential to also be vaccine candidates or targets for therapeutic drugs.

Taken together, the findings reported in this study suggest that Coccidioidal protein expression in vivo is distinct from Coccidioidal protein expression in vitro amongst its two growth phases in each of 3 different culture media. Furthermore, these findings form a foundation upon which to select relevant biomarkers for antigen-based detection of *Coccidioides* in potentially infected patients.

## Figures and Tables

**Table 1:** Protein characteristics of the 27 biomarker candidates identified in this study.

	Protein Name	Signal Peptide (Y/N)	N-Glycan Site?	O-Glycan Site?	Gene Names	Mol. Weight (kDa)
1	Hsp20/alpha crystallin family protein	N	N	Y	CPC735_047390	24
2	Peroxisomal membrane protein	N	N	N	CPC735_024650	18
3	Cofilin	N	N	N	CISG_03898	17
4	Pyruvate decarboxylase	N	Y	Y	CPSG_03493	63
5	Uncharacterized protein CPC735_057740	N	Y	N	CPC735_057740	16
6	Uncharacterized protein CISG_02340	N	N	Y	CISG_02340	12
7	GTP-binding protein sarA	Y	Y	Y	CPC735_069720	21
8	Uncharacterized protein CIMG_09001	Y	N	N	CPAG_07918	13
9	Protein wos2	N	N	Y	CPC735_022920	21
10	Eukaryotic porin family protein	Y	N	Y	CPC735_002880	30
11	Ketol-acid reductoisomerase, mitochondrial	N	Y	Y	CPC735_067570	45
12	Uncharacterized protein CIMG_05576	Y	N	Y	CPSG_05795	57
13	Glycine-rich protein	N	Y	Y	CPC735_026500	36
14	Uncharacterized protein CIMG_00509	Y	N	Y	CPC735_057210	11
15	Urease accessory protein ureG	N	Y	Y	CISG_10193	28
16	ATP synthase subunit 5	Y	N	Y	CISG_09525	30
17	Cytochrome c oxidase polypeptide VI	N	Y	Y	CPSG_00594	18
18	Dihydrodipicolinate synthetase	N	N	N	CPSG_04876	33
19	Uncharacterized protein CISG_09979	N	Y	N	CISG_09979	13
20	ATP synthase delta chain, mitochondrial	N	Y	Y	CPC735_070430	18
21	ATP synthase subunit d, mitochondrial	N	N	Y	CPC735_012590	20
22	Uncharacterized protein CPC735_030710	N	Y	Y	CPC735_030710	21
23	Protein disulfide-isomerase	Y	Y	Y	CPC735_035440	57
24	Aha1 domain-containing protein	N	Y	Y	CPSG_01619	36
25	Flavodoxin domain containing protein	N	Y	N	CPC735_070770	22
26	NADP-dependent leukotriene B4 12-hydroxydehydrogenase	N	N	N	CPAG_08737	38
27	Fructose 1,6-bisphosphate aldolase	N	N	N	CPC735_006240	40

## Supplementary Materials

The following are available online at https://www.mdpi.com/2309-608X/6/4/365/s1. Table S1: Mean normalized spectral abundances (NSAF) and protein characteristics of the 27 biomarker candidates identified in this study; Supplementary Table S2: Full list of proteins and peptides identified in this study.
